# Understanding Biomarkers in Early-Stage Invasive Breast Cancer: Tools From the ASCO Clinical Guideline

**Published:** 2016-09-01

**Authors:** Kelley D. Mayden

**Affiliations:** Wellmont Cancer Institute, Bristol, Virginia

The American Cancer Society (2015) estimates that 231,840 new cases of invasive breast cancer will be diagnosed among females in the United States in 2016. The good news is that there has been a decline in the overall death rate from breast cancer. This decline is due, in part, to improvements in early detection and screening. In addition, the identification of the presence or absence of the estrogen receptor (ER), the progesterone receptor (PR), and the human epidermal growth factor receptor (HER2) among breast cancers has paved the way for targeted and tailored therapy, thus contributing to improvements in breast cancer outcomes and decreases in breast cancer mortality. Furthermore, the mapping of the human genome has created a new era of personalized and precision medicine that takes into account individual genetic profiles, which can further define cancer therapy with the hope of greater improvements in overall survival.

Biomarkers are tools that can be used clinically to help guide treatment decisions. A biomarker is "a biological molecule found in blood, other body fluids or tissues that is a sign of a normal or abnormal process, or of a condition or disease" (National Cancer Institute, 2016). Medical genomics has provided a number of biomarkers (see Table 1) to assist in guiding clinical decision-making in breast cancer. Although the promise of biomarkers is exciting, understanding the clinical utility and validity of currently available biomarkers can be challenging for advanced practitioners (APs).

**Table 1:**
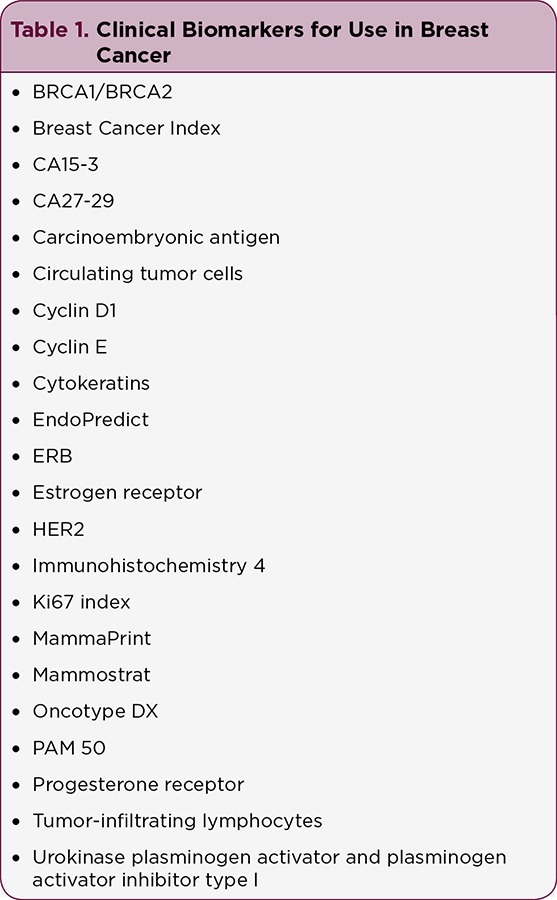
Clinical Biomarkers for Use in Breast Cancer

The American Society of Clinical Oncology (ASCO) has recently published an evidence-based clinical practice guideline that provides recommendations on the appropriate use of breast tumor biomarker assay results to guide decisions on adjuvant systemic therapy for women with early-stage invasive breast cancer and known ER/PR/HER2 status (Harris et al., 2016). The guideline was framed by the Evaluation of Genomic Applications in Practice and Prevention Working Group and addresses a number of biomarkers and gene-expression patterns (see Table 2). A panel of experts used informal consensus to frame the recommendations, and evidence was based on systematic reviews, meta-analyses, randomized controlled trials, prospective/retrospective studies, and one prospective comparative observational study published from 2006 through 2014 (Harris et al., 2016). A complete copy of the guideline is available at www.asco.org/guidelines. This article will review five of the major biomarkers found within the ASCO guideline in an attempt to acquaint APs with these available biomarkers.

**Table 2:**
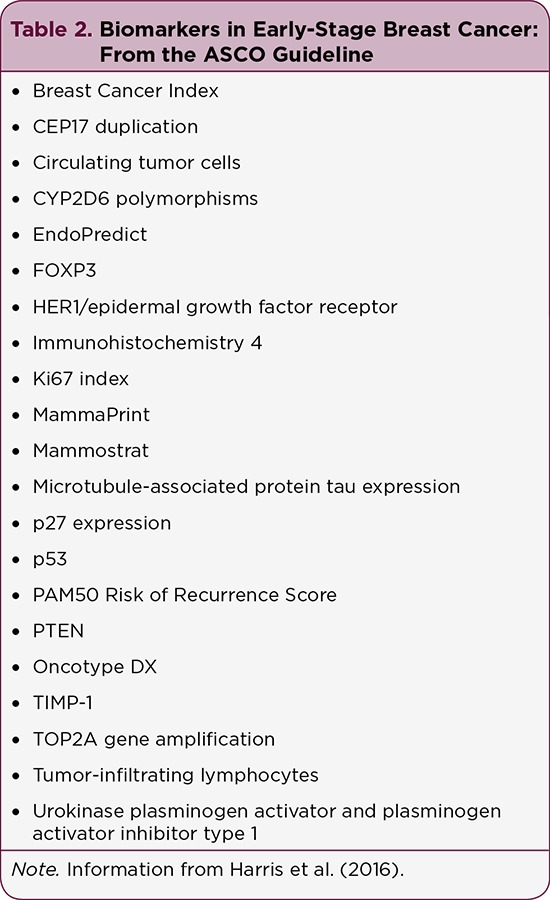
Biomarkers in Early-Stage Breast Cancer: From the ASCO Guideline

## Oncotype DX

Oncotype DX is a 21-gene polymerase chain reaction assay that has been validated to predict recurrence risk and chemotherapy benefit in hormone receptor–positive invasive breast cancer (Shak et al., 2015). The recurrence-score algorithm is based on markers of estrogen, invasion, proliferation, HER2, and reference markers (Paik et al., 2004). Oncotype DX testing impacts adjuvant treatment decisions and, in many cases, recommends against the use of systemic chemotherapy. In one study, treatment decisions were altered in 44% of patients as a result of testing (Asad et al., 2008). A separate study involving 89 assessable patients revealed a treatment decision change by 17 medical oncologists 31.5% of the time as a result of testing (Lo et al., 2010).

A prospective trial involving 10,253 women with hormone receptor–positive, HER2- negative, axillary node–negative breast cancer with tumors of 1.1 to 5.0 cm in greatest dimension used the Oncotype DX to calculate a risk score. Those with a low risk of recurrence (score of 0–10 on a scale of 0–100) were assigned to endocrine therapy alone without chemotherapy. Approximately 99.3% of node-negative, hormone receptor–positive, HER2-negative patients had no distance recurrence at 5 years after endocrine therapy alone (Sparano et al., 2015).

Genomic Health and the Surveillance, Epidemiology, and End Results (SEER) Program of the National Cancer Institute joined in an effort to electronically supplement the SEER registries with Oncotype DX results. The first report characterized breast cancer survival in node-negative hormone receptor–positive-invasive breast cancer. The 5-year breast cancer–specific survival outcomes were 99.6% in over 21,000 patients with low recurrence scores (Shak et al., 2015).

Based on high-quality evidence, ASCO strongly recommends the use of Oncotype DX to guide decisions on adjuvant therapy for patients with ER/PR-positive, HER2-negative, node-negative breast cancer. However, in patients with node-positive or HER2-positive breast cancer, ASCO does not recommend the use of Oncotype DX in clinical decision-making (Harris et al., 2016). Oncotype DX is included in the National Comprehensive Cancer Network’s (NCCN) guidelines for the treatment of breast cancer.

## EndoPredict

EndoPredict is an RNA-based assay of eight disease-relevant genes and three reference genes as expressed within tumor tissue. It is used to predict the risk of metastases for patients with ER-positive, HER2-negative breast cancer treated with endocrine therapy alone. It incorporates genomics, tumor size, and nodal status (Muller et al., 2013). Together, these factors result in a score known as the EPclin.

Patients with an EPclin score of < 3.3 were classified as low risk for distant recurrence, whereas patients with an EPclin score ≥ 3.3 were stratified as high risk for distant recurrence (Dubsky et al., 2013). Scores were validated independently in patients from two large randomized phase III Austrian Breast and Colorectal Cancer Study Groups (ABCSG-6 and ABCSG-8; Filipits et al., 2011). The EPclin result serves to guide providers as they make decisions about the addition of systemic chemotherapy.

The formal ASCO recommendation for use of EndoPredict to help guide decisions on adjuvant systemic chemotherapy is moderate and intended for use in patients with ER/PR-positive, HER2-negative, node-negative breast cancer. The ASCO panel does not recommend the use of EndoPredict in patients with ER/PR-positive, HER2-negative, node-positive breast cancer. Additionally, for patients with HER2-positive breast cancer, the assay is not recommended for use.

## PAM50

The PAM50 is a validated reverse transcription polymerase chain reaction test based on a panel of 50 genes (Nielsen et al., 2014). Performed on formalin-fixed, surgically resected breast cancer tissue, the test serves to classify a tumor into one of four subtypes (luminal A, luminal B, HER2-enriched, and basal-like), which have been shown to have prognostic value in both untreated and treated patients (Nielsen et al., 2014).

Incorporating this technology is NanoString’s Prosigna Breast Cancer Prognostic Gene Signature Assay. In addition to identifying the breast cancer subtype, the test generates a risk of recurrence score and a risk category. The score value is between 0 and 100 and correlates with the probability of distant recurrence within 10 years. The test is indicated for use in postmenopausal women with breast cancer with stage I/II, node-negative or stage II, node-positive (one to three nodes), hormone-positive disease (NanoString Technologies, 2013).

While providing a strong recommendation for use in patients with ER/PR-positive, HER2-negative, node-negative breast cancer, ASCO does not advocate the use of PAM50-based testing as a guide for decisions on adjuvant systemic therapy in women with ER/PR-positive, HER2-negative, node-positive breast cancer. In addition, ASCO does not incorporate this type of testing in patients with HER2-positive breast cancer or those with triple-negative breast cancer.

Although the prognosis for women with early-stage, hormone receptor–positive breast cancer treated with 5 years of endocrine therapy remains good, the risk of distant recurrence is still a concern for all. This concern is what led to the exploration of the concept and currently accepted clinical practice of extended endocrine therapy.

The Adjuvant Tamoxifen: Longer Against Shorter (ATLAS) trial found that for women with ER-positive disease, continuing tamoxifen to 10 years rather than stopping at 5 years produced a further reduction in recurrence and mortality, particularly after year 10 (Davies et al., 2013). The Adjuvant Tamoxifen: To Offer More (aTTom) trial revealed that in women with ER-positive disease, continuing tamoxifen to year 10 rather than just to year 5 produced further reductions in recurrence, from year 7 onward, and breast cancer mortality after year 10 (Gray et al., 2013). A group of postmenopausal (n = 5,187) women who had completed tamoxifen were randomized to an additional 5 years of endocrine therapy with letrozole vs. placebo. The investigators concluded that letrozole after tamoxifen was well tolerated and improved both disease-free and distant disease-free survival but not overall survival, except in node-positive patients (Goss et al., 2005).

Recently, Goss et al. (2016) published results of 1,918 women enrolled in a double-blind, placebo-controlled trial assessing the effect of the extended use of letrozole for an additional 5 years. The authors concluded that extending treatment with an adjuvant aromatase inhibitor to 10 years resulted in significantly higher rates of disease-free survival and a decreased incidence of contralateral breast cancer. The rate of overall survival with the aromatase inhibitor was not superior to that with placebo (Goss et al., 2016).

Although such findings clearly support a role for extended therapy, deciding which patients may benefit most from extended therapy is an important part of advanced practice. As always, the risk of any therapy must be balanced against it benefits. Risks of extended endocrine therapy include an increased risk of endometrial cancer with continued tamoxifen, hot flashes, deep vein thrombosis, ischemic heart disease, loss of bone mass, cognitive dysfunction, and vaginal dryness (Burstein et al., 2014). The Breast Cancer Index (BCI) may be of value in helping to decide which patients are appropriate candidates for extended endocrine therapy.

## Breast Cancer Index

The BCI is an 11–gene-expression–based assay that embodies two distinct predictors; a 2-gene endocrine sensitivity marker based on the ratio of *HOXB13* and *IL17BR* expression; and a 5-gene predictor (the molecular grade index), which recapitulates tumor grade and/or proliferation (Sgroi et al., 2016; Zhang et al., 2013). The assay is performed on formalin-fixed paraffin-embedded tissue and provides a high-risk or low-risk score for both predictors; it is designed to assist providers with the clinical decision to stop or extend adjuvant endocrine therapy. The BCI has been validated as prognostic for early and late distant recurrences and is predictive of adjuvant and extended adjuvant hormonal benefit in patients with early-stage, hormone receptor–positive, lymph node–negative breast cancer (Ma et al., 2004; Sgroi et al., 2013; Zhang et al., 2013).

Based on their review of the evidence, ASCO imparts a recommendation for use of the BCI in patients with ER/PR-positive, HER2-negative, lymph node–negative breast cancer as a means to guide decisions about adjuvant systemic therapy. They do not recommend using the BCI in patients with node-positive disease. An informal consensus strongly recommends that the BCI not be used in patients with triple-negative breast cancer.

## MammaPrint

MammaPrint is a 70-gene prognosis signature initially reported as a strong predictor of a short interval to distant metastases in patients without tumor cells in local lymph nodes at diagnosis (van’t Veer et al., 2002). Its clinical utility is in assisting providers in determining the necessity of adjuvant systemic chemotherapy.

Based on the gene profile of the tumor, patients are assigned a low-risk or high-risk result. A low-risk score indicates a patient has a 10% chance of breast cancer recurring within 10 years without any additional adjuvant therapy, either hormonal therapy or chemotherapy. A high-risk score indicates a patient has a 29% chance of breast cancer recurring within 10 years without any additional adjuvant therapy, either hormonal therapy or chemotherapy (Delahaye et al., 2013). The test has been validated in several retrospective studies and in a recent prospective clinical study for a breast cancer recurrence assay, Microarray Prognostics in Breast Cancer (RASTER; Bueno-de-Mesquita et al., 2009; Buyse et al., 2006; van de Vijver et al., 2002).

A formal recommendation for the clinical use of MammaPrint testing was not issued by ASCO in those patients with ER/PR-positive, HER2-negative, node-positive or node-negative breast cancer. Likewise, it was not recommended for use in HER2-positive or triple-negative breast cancers. The panel awaits and will examine the results of another prospective trial, MINDACT (Microarray in Node-Negative and One to Three Positive Lymph Node Disease May Avoid Chemotherapy), which may or may not change the future recommendation for MammaPrint use (Harris et al., 2016). The study is currently active but not recruiting patients. For details, visit www.ClinicalTrials.gov, study identifier NCT00433589. 

Following the printing of the ASCO guideline, Piccart et al. (2016) presented an abstract of the primary analysis from the MINDACT trial at the annual meeting of the American Association for Cancer Research; it concluded that the use of MammaPrint among clinically high-risk patients resulted in a 46% reduction in the use of chemotherapy.

## Conclusion

These biomarkers represent some of the most researched and recognized in clinical practice, along with several other biomarkers for use in patients with early-stage breast cancer. Breast cancer treatment continues to evolve and will no doubt be permanently influenced not only by the microscope but the molecular profile as well. As a result, APs need a working knowledge of all available biomarkers to appropriately apply the information they impart to each individual patient’s case.

The ASCO guideline serves as an evidence-based, relevant blueprint for the incorporation of biomarkers into clinical practice. The guideline also clarifies which biomarkers the panel considers are not of clinical utility in guiding the choice for adjuvant therapy (see Table 3). It is strongly recommended that APs review the guideline in detail. An additional resource for information on the use of biomarkers in breast cancer includes the NCCN Biomarker Compendium.

**Table 3:**
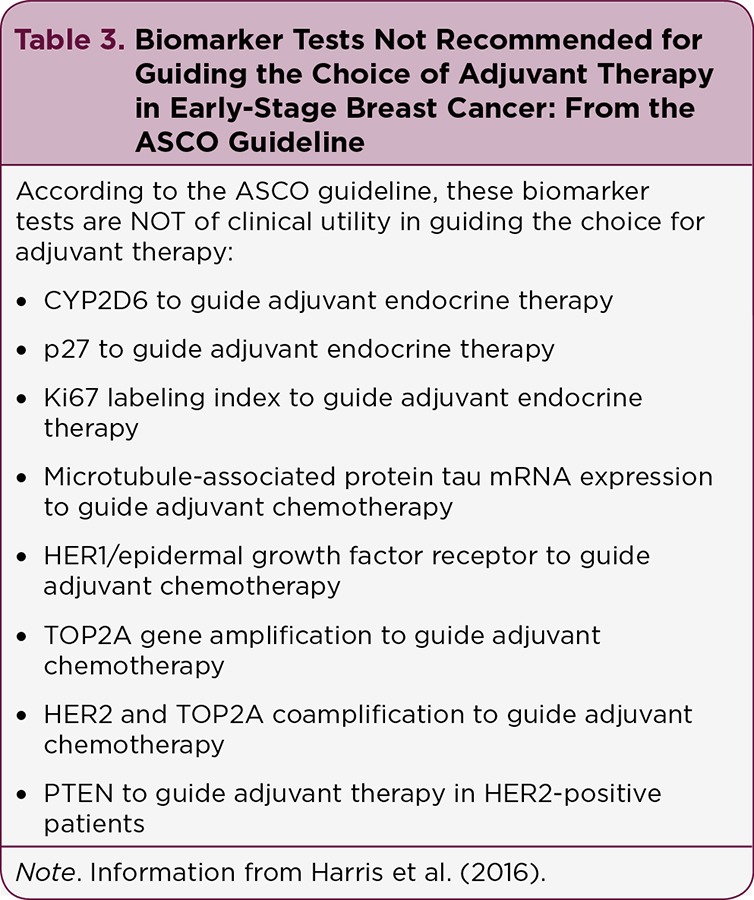
Biomarker Tests Not Recommended for Guiding the Choice of Adjuvant Therapy in Early-Stage Breast Cancer: From the ASCO Guideline
